# Evaluation of blood perfusion using laser doppler flowmetry during endoscopic lumbar sympathectomy in patients with plantar hyperhidrosis: a retrospective observational study

**DOI:** 10.1038/s41598-022-14778-7

**Published:** 2022-07-06

**Authors:** Yea-Chan Lee, Young Kyung You, Sungsoo Lee, Duk Hwan Moon, Ji-Won Lee

**Affiliations:** 1 Department of Family Medicine, Yonsei University College of Medicine, Severance Hospital, 50-1, Yonsei-ro, Seodaemun-gu, Seoul, 03722 Republic of Korea; 2 The 3rd Air and Missile Defense Brigade, Air and Missile Defense Command, Republic of Korea Air Force, Seoul, Republic of Korea; 3 Department of Family Medicine, Gangnam Severance Hospital, Yonsei University College of Medicine, 211 Eonju-ro, Gangnam-gu, Seoul, 06273 Republic of Korea; 4 Department of Thoracic and Cardiovascular Surgery, Gangnam Severance Hospital, Yonsei University College of Medicine, 211 Eonju-ro, Gangnam-gu, Seoul, 06273 Republic of Korea; 5 Department of Family Medicine, Severance Hospital, Yonsei University College of Medicine, 50-1, Yonsei-ro, Seodaemun-gu, Seoul, 03722 Korea

**Keywords:** Diseases, Medical research

## Abstract

The purpose of this study is to evaluate whether monitoring the changes of skin blood flow may be effective in assessing blood perfusion during endoscopic lumbar sympathectomy (ELS) in patients with plantar hyperhidrosis. In this study, a total of 30 patients who underwent surgical treatment for plantar hyperhidrosis at the Department of Thoracic and Cardiovascular Surgery in Yonsei University Gangnam Severance Hospital, Seoul, Korea, between July 2020 and December 2020, were retrospectively analyzed. Sympathetic denervation was performed on the third lumbar ganglion, and intraoperative laser doppler flowmetry (LDF) was used to detect the lumbar sympathetic chain accurately. We observed an abrupt increase of peripheral blood flow after sympathetic denervation, and the median percent changes of perfusion unit were 173.27 (inter-quartile range, IQR 195.48) and 392.98 (IQR 597.27) for the left and right sympathectomies, respectively. This study demonstrated the efficacy of monitoring skin blood flow via LDF during ELS. This result suggests that exact detection of blood flow using LDF is essential for improving the accuracy of ELS by checking the perfusion site on the sole in patients with plantar hyperhidrosis.

## Introduction

Primary hyperhidrosis is defined as excessive sweat production in multiple areas throughout the body. It has been shown that as high as 50% of cases involve the sole of the feet, or also known as plantar hyperhidrosis^1^. Hyperhidrosis can cause frustration, social debilitation, and anxiety in patients^2^. Therefore, effective treatment can improve the quality of life, especially in patients with plantar hyperhidrosis^2,3^. Although there are several treatment modalities for plantar hyperhidrosis^3^, there are no definite solutions which have been established appropriately^4^. Previous studies suggest that surgical sympathetic denervation may play a significant role in treating moderate-to-severe plantar hyperhidrosis with lower morbidity and fewer side effects than other therapeutic options^5–7^.

During surgical sympathetic denervation, it is important for a surgeon to assess the exact anatomical distribution of the sympathetic chain for higher accuracy of the procedure and it can also lead to minimizing potential complications, including post-sympathectomy neuralgia and sexual dysfunction^3^. To detect the target level of denervation, several intraoperative physiologic monitoring tools, such as infrared thermography, have conventionally been used. Recently, the measurement of skin blood flow via laser doppler flowmetry (LDF) has been used to provide a more rapid and precise indicator for sympathetic denervation^8^. According to several studies, researchers found that skin blood flow was rapidly decreased during the endoscopic thoracic sympathectomy and increased right after the successive procedure^9–11^.

A recent study suggested that measuring skin blood flow via LDF is superior to checking skin temperature in monitoring denervation quantitatively and qualitatively during sympathectomy^8^. Measuring the intraoperative fingertip temperature has several obstacles, including undetectable changes in minimally invasive endoscopic thoracic sympathectomy, slow reflection to surgical ablation, and hide-effect of anesthetics on a redistribution of heat; considering these limitations, measuring the intraoperative skin blood flow via LDF has been considered to a better intraoperative choice^8,12^. However, there is limited information on the intraoperative assessment of skin blood flow in plantar hyperhidrosis, despite the rising number of surgical interventions. Hence, the purpose of this study is to evaluate the efficacy of intraoperative monitoring of skin blood flow using LDF for assessing blood perfusion and finding the exact anatomical target level of the sympathetic chain during endoscopic lumbar sympathectomy (ELS) in patients with plantar hyperhidrosis.

## Methods

### Patients

Patients over the age of 19 years who underwent surgical treatment for plantar hyperhidrosis at the Department of Thoracic and Cardiovascular Surgery in Yonsei University Gangnam Severance Hospital, Seoul, Korea, between July 2020 and December 2020, were retrospectively analyzed. A total of 34 patients were identified. Four patients were excluded due to missing data regarding perfusion unit measurement. Therefore, a total of 30 patients were included in our study. And we performed percutaneous laser blood flow monitoring during ELS for these participants. We examined preoperative physical examination, laboratory tests, and medical records on demographic data and clinical status. Additionally, all patients were given self-administered questionnaires about the severity and anatomical distribution of hyperhidrosis, quality of life, social history, and health-related behavioral habits. This study was approved by the Institutional Review Board of Gangnam Severance Hospital (IRB #3-2021-0179).

### Data collection

Socio-demographic data were collected and medical examination, including severity and affected regions of hyperhidrosis, comorbid conditions, and preoperative laboratory tests, were performed in the outpatient department within one week before surgery. All patients were hospitalized one day before the operation, and the body mass index (BMI), waist circumference, and vital signs, including systolic blood pressure (SBP), diastolic blood pressure (DBP), heart rate, pulse rate, and body temperature, were measured on the day of hospitalization. Body weight (kg), height (m), and waist circumferences (cm) were measured by a body composition analyzer, InBody 720; and BMI was calculated as the ratio of body weight (kg) to the square of height (m). Before checking blood pressure and heart rate, each patient took a rest for at least 5 min. The cuff of the electronic blood pressure monitor was placed on the right upper arm of the patient above the antecubital fossa. Body temperature was measured at the tympanic membrane by electronic thermometers. Serum laboratory findings were measured with at least 8 h fasting state. Complete blood count and differential count were measured by an automatic blood cell analyzer (ADIVA 120, Bayer, NY, USA). High sensitivity C-reactive protein was measured using a biochemistry analyzer (Roche Cobas Analyzer, Germany). Serum total cholesterol, triglycerides, high density lipoprotein cholesterol, low density lipoprotein cholesterol, and fasting plasma glucose were measured with an automated chemistry analyzer (Hitachi 7600, Tokyo, Japan). Serum thyroid function test, including triiodothyronine, free thyroxine, and thyroid-stimulating hormone was performed using an immunoassay (Roche Diagnostics, Mannheim, Germany).

### Surgical technique of endoscopic lumbar sympathectomy

**Figure 1:**
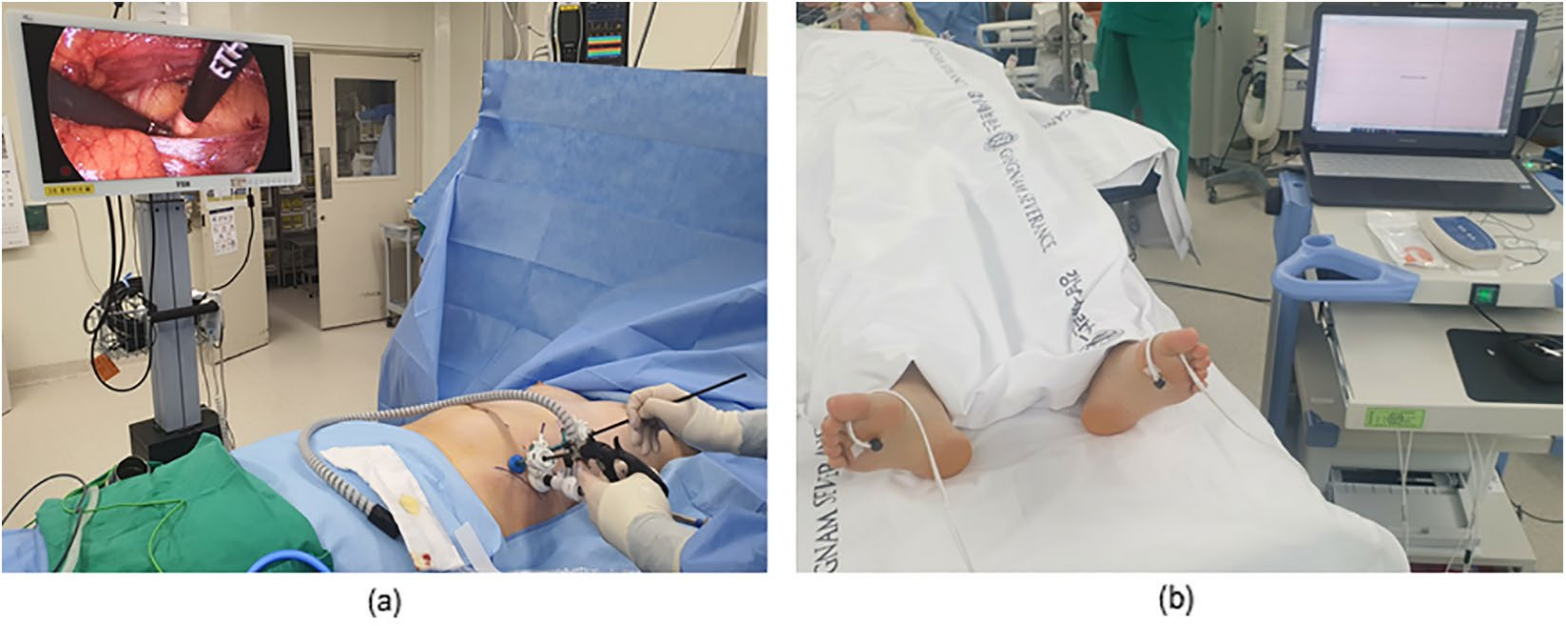
Performance of endoscopic lumbar sympathectomy. (**a**) The patient was lying down in supine position during the surgical procedure under general anesthesia. Three trocars were inserted on the lateral abdominal wall between the rib cage and hip bone. (**b**) A probe of laser doppler flowmetry was attached on the center of each sole of the patient by a holder using double-sided adhesive tape.

Surgery was performed under general anesthesia and endotracheal intubation in supine position. Three trocar insertion sites were used on each side of lateral abdominal wall between the rib cage and hip bone (Fig. 1a). First, a 15 mm skin incision was made on the middle of the lateral abdominal wall (from the umbilicus, the point made by a perpendicular line between the transverse line and lateral abdominal wall) by splitting the external oblique muscle, internal oblique muscle, and transversalis muscle (in this order). Retreoperitoneal space was considered to be established once the retroperitoneal fat was exposed. Next, a 12 mm trocar (Covidien®, Spacemaker TM Pro) with a ‘space maker’ function was inserted. The ballooning allows for a medial movement of the peritoneum and an expansion of the retroperitoneal space, allowing for adequate surgical field of view. After removing the 12 mm trocar and confirming the space using a finger, 5 mm ballooning trocars were inserted in both directions (one in the direction of the rib cage, and another in the direction of the hip bone). Surgery proceeded under continuous carbon dioxide inflation. To accurately detect the lumbar sympathetic chain, an LDF was used; the integrated LDF probes were attached to the soles of both feet to check the blood perfusion. After finding the third lumbar ganglion, sympathectomy via electro-cauterization (hook bovie) was performed. It was performed on the left side first, followed by the right side. All surgical procedures were recorded.

### Intraoperative monitoring

Percutaneous blood flow of patients was measured on each side of their soles by noninvasive laser doppler perfusion monitor probes (Periflux System 5000; Perimed, Stockholm, Sweden). The probe was attached by a holder using double-sided adhesive tape so that it was placed on each center of plantar regions (Fig. 1b). Laser light was applied to the skin through fiber optics, the value from the photo detector was electronically processed and the signal was converted into an arbitrary perfusion unit (PU), controlled by the PeriFlux System 5000 internal software. PU represents a relative value of the microvascular circulation, as the product of the number and velocity of moving blood cells. The percent change of PUs was calculated as the ratio of the change in PUs before and after ELS to the value of PU before ELS.

### Statistical analyses

Categorical variables in patient characteristics were presented as the numbers (percentage) and mean ± standard deviation (SD) for continuous variables. The average of percent change of perfusion unit was shown as the median ± inter-quartile range (IQR). P values less than 0.05 were considered to indicate statistical significance.

### Ethical approval

This study followed the ethical guidelines by the 1975 Declaration of Helsinki. Procedures of the study were approved by the Institutional Review Board of Gangnam Severance Hospital.

### Consent to participate

All the participants were provided and agreed informed consent before the investigation. All data from this retrospective observational study were obtained through an anonymized dataset.

### Consent to publication

Informed consent for publication was obtained.

## Results

The mean age of the participants was 28.8 (± 6.8) years, and a total of 18 males (60%) and 12 females (40%) were included (Table 1). The average SBP and DBP were 122.7 (± 13.8) mmHg and 74.1 (± 10.5) mmHg, respectively. The mean values (± SD) of preoperative laboratory findings are shown in Table 1.

**Table 1:** Clinical characteristics of the study population*.

Variables	Case (N = 31)
Age (years)	28.8 ± 6.8
Sex (% male)^†^	18 (60)
BMI (kg/m^2^)	22.9 ± 3.2
SBP (mmHg)	122.7 ± 13.8
DBP (mmHg)	74.1 ± 10.5
WBC (10^3^/μL)	6.64 ± 1.70
Hb (mg/dL)	14.8 ± 1.3
PLT (10^3^/μL)	256.2 ± 50.4
hsCRP (mg/L)	0.61 ± 0.48
Total cholesterol (mg/dL)	190.2 ± 35.6
Triglycerides (mg/dL)	126.3 ± 76.3
HDL-cholesterol (mg/dL)	56.4 ± 11.2
LDL-cholesterol (mg/dL)	108.3 ± 26.8
FPG (mg/dL)	96.3 ± 10.4
T3 (ng/dL)	102.5 ± 24.0
fT4 (ng/mL)	1.30 ± 0.20
TSH (mcIU/mL)	2.89 ± 3.94

BMI, body-mass index; SBP, systolic blood pressure; DBP, diastolic blood pressure; WBC, white blood cell; Hb, hemoglobin; PLT, platelet count; hsCRP, high sensitivity C-reactive protein; HDL-cholesterol, high density lipoprotein cholesterol; LDL-cholesterol, low density lipoprotein cholesterol; FPG, fasting plasma glucose; T3, triiodothyronine; fT4, free thyroxine; TSH, thyroid-stimulating hormone. *The values are expressed as mean ± standard deviation for continuous variables or numbers (percentage) for categorical variables. ^†^Categorical variables.

**Figure 2:**
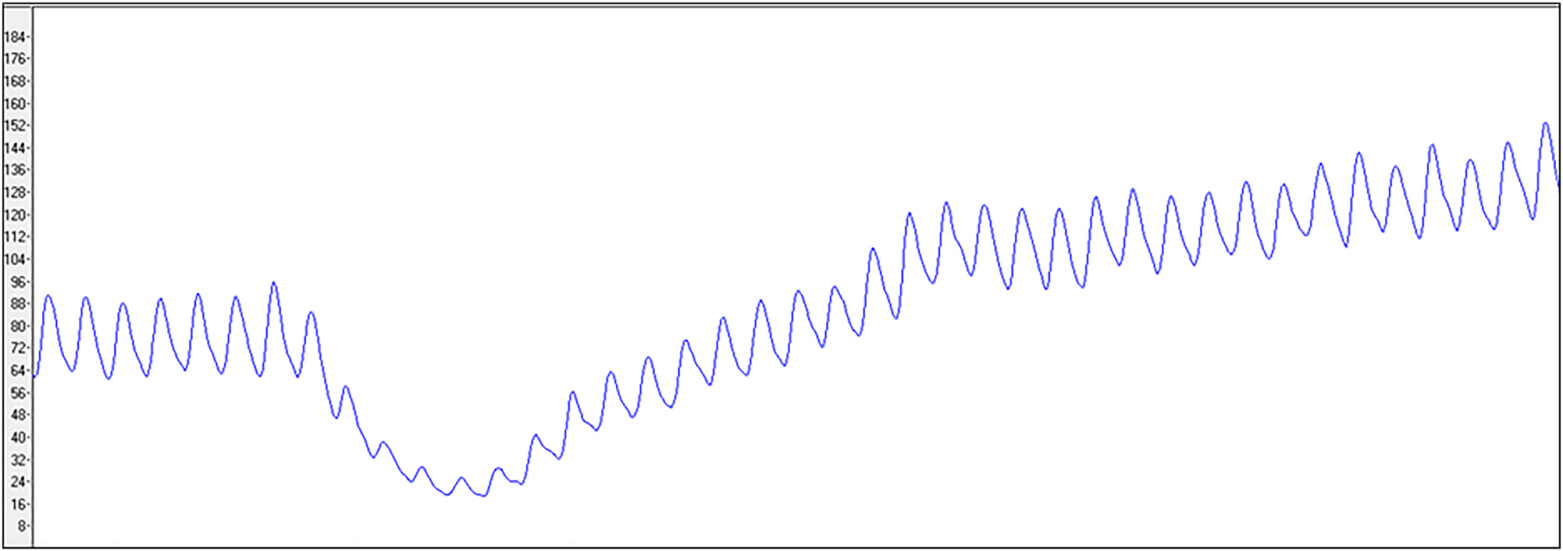
An example of graph which illustrates an actual form of intraoperative blood flow monitoring on the sole of a patient using laser doppler flowmetry during endoscopic lumbar sympathectomy. Peripheral blood flow abruptly decreased right after electro-cauterization of the lumbar sympathetic chain, increased gradually during the sympathectomy, and remained a higher level compared to that before the procedure.

The median percent changes of perfusion unit were 173.27 (IQR 195.48) and 392.98 (IQR 597.27) for the left and right sympathectomies, respectively. Individual percent change of perfusion units was shown on Supplementary Table 1 online. Figure 2 illustrates an actual example form of intraoperative tracing of peripheral blood flow monitoring during ELS in a patient. Percutaneous blood flow of the plantar region was abruptly decreased right after starting the procedure, gradually increased during electro-cauterization of sympathetic chain, and remained at a higher level than that before the sympathectomy. In this study was demonstrated a histogram with normal distribution of the percent change of PUs, and it was shown that all patients’ data of PUs increased before and after surgery (see Supplementary Fig. 1).

## Discussion

This is the first study to examine the blood perfusion using LDF during ELS in patients with plantar hyperhidrosis. ELS is not only a complicated technique, especially with respect to difficulties in the localization at the retroperitoneal space with associated potential risks, such as sexual dysfunction, problematic bleeding, or invalid ablation, but there is also limited research on the monitoring techniques during ELS when compared with endoscopic thoracic sympathectomy in patients with palmar hyperhidrosis^13,14^. In this regard, we evaluated a real-time monitoring technique that would determine the exact target levels of the sympathetic trunks using LDF.

Based on our findings, intraoperative monitoring of skin blood flow via LDF revealed an increased mean percent change of PUs by 173.27 (195.48) and 392.98 (597.27) in the left and right sole during ELS, respectively. During the procedure, percutaneous blood flow decreased instantaneously after cauterization of the lumbar sympathetic chain, and increased abruptly right after the complete sympathectomy. As LDF represents a relative quantification of the microvascular circulation, as proportional to the flux of moving blood cells, our finding indicated that reflex vasoconstriction may occur with an increase in plantar blood flow by sympathetic ablation and subsequent vasodilator effect^15–17^.

Several studies found that sympathetic nervous system plays a key role in the management of vasomotor and sudotmotor activities of the skin, which is related to hyperhidrosis^10,18,19^. In the glabrous skin regions, such as plantar region, blockage of sympathetic outflow can lead the activation of arteriovenous anastomoses, where the sympathetic vasoconstrictor nerves innervate^10^. According to Eisenach JH et al., this substantial change in the sympathetic activity can explain the immediate change in blood perfusion of the palmar region during sympathetic denervation^8^. Our recent study showed a similar change in the perfusion of plantar region during ELS, suggesting that ELS may be considered as the surgical treatment for improving perfusion on the sole of feet in patients with plantar hyperhidrosis. Moreover, increased perfusion units measured by LDF can reflect a digital vasodilatory response as the therapeutic effects of systemic sclerosis with Raynaud’s phenomenon after nitroglycerin patch treatment^20^, and LDF can be used for objectively categorizing both the extent of microvascular circulatory impairment and treatment outcome in patients with thromboangiitis obliterans, known as Buerger’s disease^21^. This suggests that we can consider the increased perfusion of the plantar region observed by LDF during ELS as potential evidence that ELS may be effective in treating peripheral vascular diseases, such as SSc and TAO^20–22^.

There are several limitations to our study. First, due to the relatively small-sized sample from a single center, there can be unadjusted confounders or biases in our study. Second, we used values of percent change to estimate the percutaneous blood flow during ELS; however, we found considerable variation in the absolute values of perfusion units before and after ELS. This might lead to a detection bias; therefore, further studies are needed to adjust for any possible biases. Third, our study did not include therapeutic outcomes or compensatory hyperhidrosis as a complication of sympathectomy surgery. As the first investigation to evaluate LDF in plantar hyperhidrosis, we believe this study would lay the foundation for subsequent studies evaluating the efficacy of LDF in plantar hyperhidrosis.

Moreover, although the Periflux System 5000 laser has a module for measuring the skin temperature in addition to the PUs, our study did not include any result related to skin temperature. Since the present study mainly focused on assessing the changes in PUs, and not those of skin temperature, we did not additionally collect data on skin temperature during perioperative monitoring. Also, considering being confounded by environmental factors surrounding the patient, skin temperature would not be suitable as a predictable indicator to assess blood perfusion during ELS in patients with plantar hyperhidrosis.In spite of these limitations, our observations also have several strengths. To the best of our knowledge, this is the first study to investigate the blood perfusion of anatomical distribution of sympathetic chain quantitatively and qualitatively during sympathectomy so that verify the potential efficacy of ELS for microvascular circulation in patients with plantar hyperhidrosis. Unlike endoscopic thoracic sympathectomy for palmar hyperhidrosis, which has been studied and proven through many studies, the efficacy of ELS for plantar hyperhidrosis remains unclear. Further randomized controlled trial investigations and larger-sized sample studies are needed to better understand the mechanism and prove the efficacy of ELS in plantar hyperhidrosis. Moreover, ELS is a very challenging surgery due to the retroperitoneal approach used in the procedure, and it is difficult for the surgeons to find the accurate anatomical site, which may cause plantar hyperhidrosis. In this aspect, our study shows that LDF can be considered as a useful intraoperative guiding tool for identification of the target nerve.

In conclusion, our study found that the perfusion units evaluated by LDF significantly increased during ELS in patients with plantar hyperhidrosis. This study demonstrates the efficacy of monitoring skin blood flow via LDF during ELS and suggests that appropriate monitoring of blood flow using LDF is useful for improving the accuracy of ELS by checking the perfusion site on the sole. Nonetheless, subsequent studies are needed to better understand the mechanism and further evaluate the efficacy of ELS in plantar hyperhidrosis.

## Supplementary Information

Supplementary Information 1.

Supplementary Information 2.

## Data Availability

The data that were used in this study are available upon reasonable request from the corresponding author, D.H.M.
