# Supplementary material for: Evaluation of blood perfusion using laser doppler flowmetry during endoscopic lumbar sympathectomy in patients with plantar hyperhidrosis: a retrospective observational study
Source: Sci Rep. 2022 Jul 6;12:11456. doi: 10.1038/s41598-022-14778-7 (PMC9259612; doi:10.1038/s41598-022-14778-7)
Supplement: Supplementary file 1 — Supplementary Information 1. [file 41598_2022_14778_MOESM1_ESM.pdf]

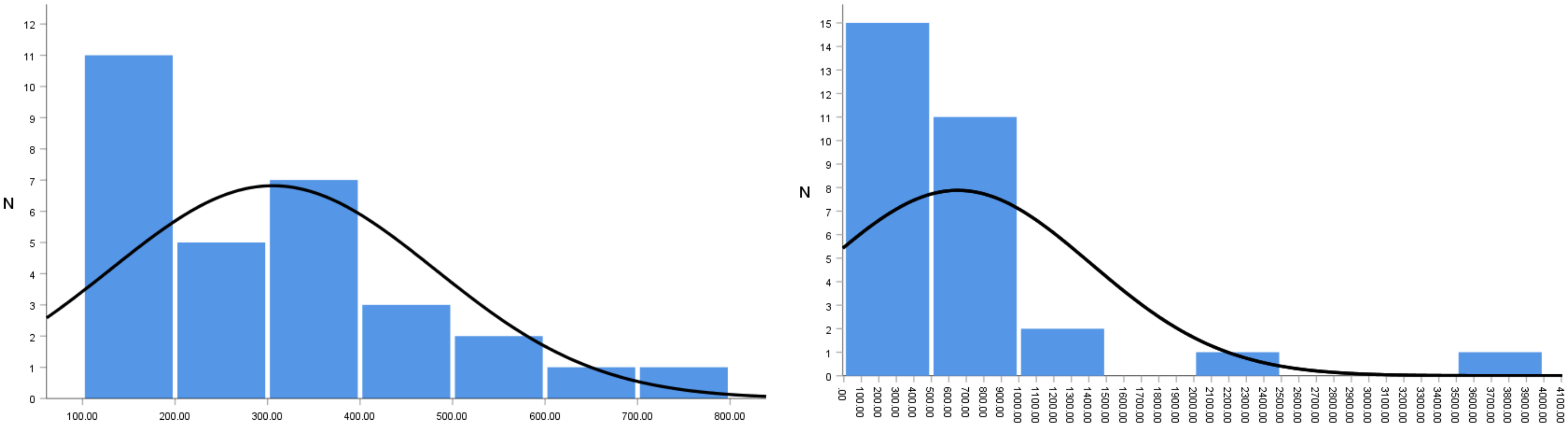

(a) (b)

Supplementary figure 1. Histogram with normal distribution of the percent change of perfusion units(PUs) of the patients. (a) Distribution of the percent change of PUs in left plantar regions (b) Distribution of the percent change of PUs in right plantar regions.
