# Supplementary material for: Evaluation of blood perfusion using laser doppler flowmetry during endoscopic lumbar sympathectomy in patients with plantar hyperhidrosis: a retrospective observational study
Source: Sci Rep. 2022 Jul 6;12:11456. doi: 10.1038/s41598-022-14778-7 (PMC9259612; doi:10.1038/s41598-022-14778-7)
Supplement: Supplementary file 2 — Supplementary Information 2. [file 41598_2022_14778_MOESM2_ESM.pdf]

Supplementary Table 1. Individual percent change of perfusion units on the left and right side of soles of the patients.

N	PU_L_Pre	PU_L_Post	PU_L_Change	PU_R_Pre	PU_R_Post	PU_R_Change
1	56.22	58.38	3.84	47.93	52.47	9.46
2	58.1	67.73	16.57	153.68	160.55	4.47
3	9.87	33.07	235.15	6.83	23.57	244.95
4	6.93	55.39	698.92	1.8	66.02	3570
5	24.54	53.29	117.2	130.02	141.8	9.06
6	11.58	80.9	598.72	13.16	92.64	603.78
7	26.48	40.22	51.87	5.09	66.76	1211.74
8	10.42	28.69	175.3	9.09	52.28	475.4
9	12.37	60.54	389.58	16.77	123.25	634.97
10	44.72	58.16	30.06	88.04	163.82	86.08
11	5.43	16.99	212.8	7.36	8.38	13.85
12	40.1	41.86	4.38	36.14	45.21	25.09
13	48.17	84.86	76.15	40.52	148.57	266.69
14	73.16	137.08	87.36	17.82	81.71	358.61
15	44.73	88.07	96.87	22.2	66.64	200.18
16	23.41	85.64	265.83	10.5	55.39	427.35
17	6.26	25.45	306.56	4.67	38.06	715.18
18	11.92	60.14	404.59	5.11	37.76	638.49
19	72.3	121.27	67.74	55.49	61.73	11.23
20	26.38	34.87	32.18	70.66	72.92	3.2
21	83.98	100.78	20	4.6	24.52	433.37
22	26.83	72.79	171.24	8.81	126.86	1340.07
23	34.59	92.49	167.4	22.16	70.22	216.83
24	25.46	111.78	338.99	107.25	119.83	11.73
25	15.8	89.98	469.54	5.64	31.43	457.48
26	6.24	20.73	232.33	1.58	39.23	2375.87
27	25.62	93.21	263.78	9.69	73.67	660.01
28	42.5	105.77	148.88	17.21	92.83	439.32
29	23.34	84.67	262.76	13.22	113.72	760.23
30	26.7	86.08	222.39	20.13	83.04	312.5

Abbreviations: PU_L_Pre, Measured value of perfusion unit on the left sole before ELS; PU_L_Post, Measured value of perfusion unit on the left sole after ELS; PU_Change_L, Percent change of perfusion units of the left sole before and after ELS; PU_R_Pre, PU_R_Post, and PU_Change_R, respectively same as those on the right sole.
